# Association of glial fibrillary acid protein, Alzheimer's disease pathology and cognitive decline

**DOI:** 10.1093/brain/awae211

**Published:** 2024-06-28

**Authors:** Débora E Peretti, Cecilia Boccalini, Federica Ribaldi, Max Scheffler, Moira Marizzoni, Nicholas J Ashton, Henrik Zetterberg, Kaj Blennow, Giovanni B Frisoni, Valentina Garibotto

**Affiliations:** Laboratory of Neuroimaging and Innovative Molecular Tracers (NIMTlab), Geneva University Neurocentre and Faculty of Medicine, University of Geneva, Geneva 1205, Switzerland; Laboratory of Neuroimaging and Innovative Molecular Tracers (NIMTlab), Geneva University Neurocentre and Faculty of Medicine, University of Geneva, Geneva 1205, Switzerland; Laboratory of Neuroimaging of Aging (LANVIE), University of Geneva, Geneva 1205, Switzerland; Geneva Memory Centre, Department of Rehabilitation and Geriatrics, Geneva University Hospitals, Geneva 1205, Switzerland; Division of Radiology, Geneva University Hospitals, Geneva 1205, Switzerland; Biological Psychiatry Unit, IRCCS Istituto Centro San Giovanni di Dio Fatebenefratelli, Brescia 25125, Italy; Centre for Age-Related Medicine, Stavanger University Hospital, Stavanger 4011, Norway; Department of Psychiatry and Neurochemistry, Institute of Neuroscience and Physiology, The Sahlgrenska Academy at the University of Gothenburg, Mölndal 413 90, Sweden; King's College London, Institute of Psychiatry, Psychology & Neuroscience, Maurice Wohl Clinical Neuroscience Institute, London SE5 9RX, UK; Mental Health & Biomedical Research Unit for Dementia, Maudsley NIHR Biomedical Research Centre, London SE5 8AF, UK; Mental Health & Biomedical Research Unit for Dementia, Maudsley NIHR Biomedical Research Centre, London SE5 8AF, UK; Department of Neurodegenerative Disease, UCL Institute of Neurology, London WC1E 6BT, UK; Department of Neurodegenerative Disease, UCL Institute of Neurology, London WC1N 3BG, UK; Clinical Neurochemistry Laboratory, Sahlgrenska University Hospital, Mölndal 413 45, Sweden; Hong Kong Centre for Neurodegenerative Diseases, Clear Water Bay, Units 1501–1502, Hong Kong 1512–1518, China; Wisconsin Alzheimer’s Disease Research Centre, University of Wisconsin School of Medicine and Public Health, University of Wisconsin-Madison, Madison, WI 53792, USA; Department of Psychiatry and Neurochemistry, Institute of Neuroscience and Physiology, The Sahlgrenska Academy at the University of Gothenburg, Mölndal 413 90, Sweden; Clinical Neurochemistry Laboratory, Sahlgrenska University Hospital, Mölndal 413 45, Sweden; Paris Brain Institute, ICM, Pitié Salpêtrière Hospital, Sorbonne University, Paris 75013, France; Neurodegenerative Disorder Research Centre, Division of Life Sciences and Medicine, and Department of Neurology, Institute on Aging and Brain Disorders, University of Science and Technology of China and First Affiliated Hospital of USTC, Hefei 230001, China; Laboratory of Neuroimaging of Aging (LANVIE), University of Geneva, Geneva 1205, Switzerland; Geneva Memory Centre, Department of Rehabilitation and Geriatrics, Geneva University Hospitals, Geneva 1205, Switzerland; Laboratory of Neuroimaging and Innovative Molecular Tracers (NIMTlab), Geneva University Neurocentre and Faculty of Medicine, University of Geneva, Geneva 1205, Switzerland; Division of Nuclear Medicine and Molecular Imaging, Geneva University Hospitals, Geneva 1205, Switzerland; Centre for Biomedical Imaging, University of Geneva, Geneva 1205, Switzerland

**Keywords:** neurofibrillary tau tangles, Alzheimer’s disease biomarkers, glial fibrillary acidic protein, cognitive decline, positron emission tomography

## Abstract

Increasing evidence shows that neuroinflammation is a possible modulator of tau spread effects on cognitive impairment in Alzheimer's disease. In this context, plasma levels of the glial fibrillary acidic protein (*GFAP*) have been suggested to have a robust association with Alzheimer's disease pathophysiology. This study aims to assess the correlation between plasma *GFAP* and Alzheimer's disease pathology, and their synergistic effect on cognitive performance and decline.

A cohort of 122 memory clinic subjects with amyloid and tau PET, MRI scans, plasma *GFAP* and Mini-Mental State Examination (MMSE) was included in the study. A subsample of 94 subjects had a follow-up MMSE score at ≥1 year after baseline. Regional and voxel-based correlations between Alzheimer's disease biomarkers and plasma *GFAP* were assessed. Mediation analyses were performed to evaluate the effects of plasma GFAP on the association between amyloid and tau PET and between tau PET and cognitive impairment and decline.

*GFAP* was associated with increased tau PET ligand uptake in the lateral temporal and inferior temporal lobes in a strong left-sided pattern independently of age, sex, education, amyloid and *APOE* status (β = 0.001, *P* < 0.01). The annual rate of MMSE change was significantly and independently correlated with both GFAP (β = 0.006, *P* < 0.01) and global tau standardized uptake value ratio (β = 4.33, *P* < 0.01), but not with amyloid burden. Partial mediation effects of *GFAP* were found on the association between amyloid and tau pathology (13.7%) and between tau pathology and cognitive decline (17.4%), but not on global cognition at baseline.

Neuroinflammation measured by circulating *GFAP* is independently associated with tau Alzheimer's disease pathology and with cognitive decline, suggesting neuroinflammation as a potential target for future disease-modifying trials targeting tau pathology.

## Teaser

Peretti *et al.* show that a circulatory marker of neuroinflammation—glial fibrillary acidic protein—is associated with tau pathology in lateral temporal and frontal regions in patients with Alzheimer's disease, independent of amyloid load. Neuroinflammation appears to modulate the association between amyloid and tau biomarkers.

**See Pelkmans and Gispert (https://doi.org/10.1093/brain/awae354) for a scientific commentary on this article.**

## Introduction

Alzheimer's disease is a neurodegenerative disorder biologically defined by the presence of amyloid-β plaques and hyperphosphorylated tau protein deposition.^1^ PET is an imaging technique that allows for the *in vivo* visualization and quantification of Alzheimer's disease pathology.^2^ Furthermore, it also allows not only for the discrimination of Alzheimer's disease from other neurodegenerative disorders,^3,4^ but also for the staging of Alzheimer's disease based on the characteristic distribution of pathology in the brain.^5,6^ More specifically, the spatial distribution of tau aggregates has been linked to cognitive impairment and neurodegeneration.^5,7,8^

However, in addition to these established Alzheimer's disease biomarkers, studies have shown that neuroinflammation coexists with characteristic Alzheimer's disease pathology.^9,10^ In particular, astrocyte reactivity is commonly found enclosing amyloid pathology in Alzheimer's disease patients.^11,12^ This association is so well established that the National Institute on Ageing and the Alzheimer's Association (NIA-AA) is proposing revised criteria for diagnosis and staging of Alzheimer's disease, in which amyloid and tau pathology remain as the main biomarkers for disease identification, but neuroinflammation is now introduced, together with neurodegeneration, as a staging and prognosis biomarker.^13,14^

Although astrocyte reactivity has been related mainly to amyloid pathology,^12^ studies have also suggested that neuroinflammation drives propagation of tau pathology in the brain,^15,16^ thereby following the stereotyped spread in Braak stages.^17^ Although an association between neuroinflammation and tau pathology is known, additional investigation in settings closer to clinical routine are required for the perspective of a successful clinical implementation of biomarkers of neuroinflammation.

The neuroinflammatory response caused by Alzheimer's disease pathology can be assessed through the circulatory marker glial fibrillary acidic protein (*GFAP*).^12,18^*GFAP* expression measured in plasma is used for the *in vivo* identification of astroglia, and an increase of this marker is a typical indication of the presence of pathology in the CNS.^19,20^ Furthermore, plasma *GFAP* levels have been suggested to be a sensitive biomarker for detection of reactive astrogliosis.^21-23^ Beyond its link with neurodegenerative disorders, previous studies have also shown that *GFAP* is associated with deficits and decline in several cognitive domains.^24,25^ Consequently, the NIA-AA has included *GFAP* as a staging biomarker for neuroinflammation in the abovementioned revised criteria.^13^

Previous studies have shown that plasma *GFAP* levels are associated with Alzheimer's disease pathology measured in CSF^12,21,26,27^ and plasma^21,26,28^ and by neuroimaging.^12,29^ More specifically, *GFAP* has been suggested to play a role in the association between amyloid pathology and early deposition of neurofibrillary tau tangles.^26^ Moreover, *GFAP* has been shown to predict conversion from mild cognitive impairment to Alzheimer's disease dementia.^27^

The aim of this study was to investigate further the association between Alzheimer's disease pathology (i.e. amyloid and tau accumulation) measured through PET imaging and plasma *GFAP* in a memory clinic cohort. Furthermore, the correlation between *GFAP* and cognitive performance and decline was also assessed. Finally, given that neuroinflammation and Alzheimer's disease pathology have been suggested to be closely related, a mediation analysis of the effect of *GFAP* in the association between amyloid and tau, and the association between tau and cognitive performance and decline was studied.

## Materials and methods

### Subjects

A cohort of 122 subjects who consulted the Memory Clinic of the Geneva University Hospitals (HUG, Geneva, Switzerland) was included in this study. Each subject underwent work-up at the memory clinic, including clinical and neurological assessment, neuropsychological testing and 3D T_1_ MRI. Additional procedures, such as amyloid PET, tau PET and blood sampling, were performed if deemed clinically useful or in the context of other research projects. Subjects were classified clinically as cognitively unimpaired (CU), mild cognitive impairment (MCI)^30^ or dementia.^31^ Inclusion criteria were as follows: (i) amyloid and tau PET imaging performed within 12 months of each other (average 4 ± 6 months); (ii) 3D T_1_ MRI scans performed within 12 months from tau PET images (average 4 ± 8 months); (iii) neuropsychological assessment with at least one Mini-Mental State Examination (MMSE) performed within 12 months of tau PET imaging (average 3 ± 5 months); and (iv) plasma *GFAP* levels assessed within 12 months from tau PET (average 2 ± 8 months).

A subsample of 94 subjects was included who had a follow-up neuropsychological assessment including at least MMSE scores after ≥12 months after baseline (average 27 ± 15 months). The annual rate of MMSE score change was calculated, and cognitive decline was defined as an average annual rate of MMSE change of one point per year.^32^

The local review board (Cantonal Commission of Research Ethics, Geneva, Switzerland) approved the studies, which were conducted in concordance with the principles of the Declaration of Helsinki and International Conference on Harmonisation Guidelines on Good Clinical Practice. All subjects or their relatives provided voluntary written informed consent to share their data for research purposes.

### Imaging acquisition and processing

MRI examinations were performed at the HUG's Division of Radiology. 3D T_1_ images were acquired using a Magnetom Skyra 3 T scanner (Siemens Healthineers) equipped with a 64-channel head coil and were acquired in concordance with IMI pharmacog WP5/European ADNI sequences and published procedures.^33^ A field of view of 256 mm, 0.9–1 mm slice thickness, 1819–1930 ms repetition time, 2.19–2.4 ms echo time, 8° flip angle and no fat suppression were used.

PET imaging was performed at the Nuclear Medicine and Molecular Imaging Division of the HUG. All images were acquired using a Biograph PET/CT scanner (Siemens Health Solutions), reconstructed using a 3D OSEM algorithm (four iterations, eight subsets), a 2 mm Gaussian convolution kernel, corrected for dead time, normalization, attenuation and sensitivity. All radiotracers are commercially available and were synthesized at radiopharmaceutical Good Manufacturing Practice laboratories and shipped to Geneva. For amyloid PET, 41 subjects were injected with 207 ± 23 MBq ^18^F-florbetapir, and images were acquired 40 min after intravenous administration of the radiotracer for 10 min. The remaining 81 subjects were scanned using 172 ± 18 MBq of ^18^F-flutemetamol, and images were acquired 90 min after intravenous radiotracer injection for 20 min. For tau PET, ^18^F-flortaucipir, synthesized at the Centre for Radiopharmaceutical Sciences in Villigen, Switzerland, under license from the intellectual property owner (Avid subsidiary of Lilly), was used. Subjects were injected with 207 ± 50 MBq intravenously, and images were acquired 75 min after injection for 30 min.

All images were processed at the Memory Clinic of the HUG using SPM12 (Wellcome Trust Centre for Neuroimaging, London, UK) and MATLAB R2018b v.9.5 (MathWorks Inc., Sherborn, MA, USA). Initially, 3D T_1_ MRI images were aligned to the anterior commissure–posterior commissure line. Then, they were normalized to the Montreal Neurologic Institute (MNI) space using tissue probability maps.^34^ PET images were aligned to the subject's respective MRI image and then, using the transformation matrix estimated for the MRI scans, they were transformed into the MNI space. Volumes of interest (VOIs) were defined based on the automated anatomic labelling atlas 3.^35^

Amyloid PET images were converted to standardized uptake value ratios (SUVRs) using the whole cerebellum as a reference region. The average SUVR was extracted from the Centiloid VOI and converted to Centiloid units^36-38^ in order that data from different radiotracers could be compared. A Centiloid value of 12 was used to define amyloid positivity (A+).^39,40^

Tau PET images were converted to SUVR values using the cerebellar crus as a reference region.^41,42^ Tau positivity was defined based on the simplified temporal–occipital classification model.^40,42^ Average SUVR was extracted based on a global set of regions (amygdala, parahippocampus, middle occipital gyrus and temporal inferior gyrus^43^) and in Braak regions (weighted averages of the following bilateral regions: Braak I/II: hippocampus; Braak III: parahippocampal gyrus, lingual gyrus and amygdala; Braak IV: inferior temporal cortex, middle temporal cortex, temporal pole, thalamus, posterior cingulate and insula; Braak V: frontal cortex, parietal cortex, occipital cortex, superior temporal cortex, precuneus, caudate nucleus and putamen; Braak VI: precentral gyrus, postcentral gyrus, paracentral gyrus and cuneus^44^).

Cortical reconstruction and volumetric segmentation of T_1_ MRI images were performed using Freesurfer (v.7, recon-all^45^). An Alzheimer's disease cortical signature (weighted average cortical thickness in the entorhinal, inferior temporal, middle temporal and fusiform VOIs) was created.^46^

### Plasma sampling and processing

Plasma samples were collected within 1 year of tau PET examination, with participants non-fasting. Blood was collected in EDTA-plasma tubes and centrifuged (2000*g*, +4°C for 10 min). After centrifugation, plasma was aliquoted into 1.5 ml polypropylene tubes (1 ml plasma in each tube) and stored at −80°C. GFAP levels were assessed using GFAP Simoa Discovery kits for HD-X (Quanterix).^12,47^

### Statistical analyses

Subjects were classified into AT profiles based on their combined amyloid and tau statuses. A Kruskal–Wallis test and Dunn’s test for multiple corrections using Benjamini–Hochberg were performed to explore differences in age, years of education, MMSE, Centiloid, global tau SUVR, composite Alzheimer's disease cortical thickness signature and plasma *GFAP* levels between groups. A χ^2^ test was used to compare sex and *APOE* carriership differences across the groups. Significant differences between baseline and follow-up MMSE scores were assessed using a paired Wilcoxon test for each group individually.

Spearman correlations between *GFAP* levels and Centiloid, global and regional Braak tau SUVR, cortical thickness and MMSE scores at baseline were calculated for the complete data and per AT profile. Regional tau SUVR correlations with *GFAP* were also computed for right and left hemispheres separately. A multivariate linear regression model to assess the association between *GFAP* levels and global tau and Centiloid was performed, correcting for age, sex, education, *APOE* carriership and cortical thickness.

A voxel-wise regression to assess the correlation between *GFAP* and tau SUVR at a voxel level was performed, controlling for age, sex, education, *APOE* carriership and Centiloid. Finally, a voxel-wise linear regression to assess the correlation between *GFAP* and amyloid SUVR (per amyloid radiotracer) was performed, controlling for age, sex, education, *APOE* carriership and global tau SUVR. The statistical threshold for voxel-based analyses was set at *P* = 0.001, family wise error (FWE)-corrected at the cluster level. A second model was run also including baseline MMSE scores as a nuisance variable.

Spearman correlations were used to assess the correlation between baseline MMSE and MMSE annual rate of change and Centiloid, global tau SUVR and *GFAP* for the complete data and by AT profiles. A multivariate linear regression model was used to assess the association between the same variables, corrected for age, sex, education, *APOE* carriership and cortical thickness. Differences in plasma *GFAP* levels between decliners and stable individuals were assessed using a Wilcoxon test for the whole cohort and by AT status.

To examine whether the associations between Centiloid and global tau SUVR were mediated by *GFAP* levels, we performed mediation analyses controlling for age, sex, education and *APOE* carriership. Additional mediation analyses were run to examine whether the association between regional Braak tau SUVR and Centiloid, the association between global tau SUVR and MMSE scores, and the association between Centiloid and MMSE scores were mediated by *GFAP* levels. Mediation analysis was also performed to test whether the relationship between global tau SUVR or Centiloid and MMSE annual rate of change was mediated by *GFAP* levels, again correcting for age, sex, education and *APOE* carriership. Bootstrapping resampling was used to estimate confidence intervals for all mediation analyses with 1000 resampling.^48^

A *P*-value of 0.05 was considered as the significance threshold for all analyses, which were performed using RStudio (version ‘Mountain Hydrangea’, R v.4.3.1). Dunn tests were performed using the package *FSA* (v.0.9.4), multilinear regression using the package *lme4* (v.1.1) and mediation analysis using the package *mediation* (v.4.5.0). Voxel-wise analysis was run in MATLAB (R2023b v.9.12) using SPM12.

## Results

### Population

Characteristics of the included cohort of subjects at baseline is shown in Table 1 per AT profile. The average age of the population was 72 ± 8 years, 61 individuals were females (50%), average education was 14 ± 4 years, MMSE score at baseline was 26 ± 4, Centiloid was 49 ± 44 units, global tau SUVR was 1.34 ± 0.34, cortical thickness was 2.70 ± 0.18 mm, and *GFAP* levels were 188.2 ± 114.4 pg/ml. Age and sex were significantly different between groups, but no significant differences were found when correcting for multiple comparisons.

**Table 1:** Demographic, cognitive and imaging characteristics and plasma *GFAP* levels at baseline of subjects included in the study

AT status	A−T− (*n* = 47)	A−T+ (*n* = 3)	A+T− (*n* = 28)	A+T+ (*n* = 44)	*P*-value
Age (years)	70 ± 8	76 ± 5	74 ± 8	74 ± 7	0.04
Sex (female/male)	23/24	3/0	8/20	27/17	0.01
Education (years)	15 ± 4	11 ± 1	15 ± 4	13 ± 4	0.14
MMSE at baseline	27 ± 2^a^	28 ± 2	27 ± 2^a^	24 ± 5^b^	<0.01
Diagnosis stage (CU/MCI/dementia/other)	21/19/2/5	2/1/0/0	5/19/4/0	1/31/12/0	<0.01
*APOE* carriership (non-carrier/carrier)	40/7	3/0	20/8	12/32	<0.01
Centiloid	−2 ± 8^b,d^	−3 ± 11^b,d^	50 ± 29^b,c^	81 ± 32^a^	<0.01
Global tau SUVR	1.14 ± 0.09^b^	1.35 ± 0.03	1.18 ± 0.11^b^	1.67 ± 0.36^a^	<0.01
Composite Alzheimer's disease cortical thickness signature (mm)	2.78 ± 0.13^a^	2.78 ± 0.18	2.70 ± 0.18	2.62 ± 0.20^b^	<0.01
Plasma *GFAP* (pg/ml)	128 ± 97^b^	170 ± 12	201 ± 115^a^	246 ± 104^a^	<0.01

Reported *P*-values result from the Kruskal–Wallis test. Dunn’s tests for *post hoc* analysis using the Benjamini–Hochberg correction for multiple comparisons were used to compare between groups. Superscript letters indicate groups showing significant differences at *post hoc* comparison: a > b, c > d. A = amyloid; CU = cognitively unimpaired; GFAP = glial fibrillary acidic protein; MCI = mild cognitive impairment; MMSE = Mini-Mental State Examination; *n* = number of subjects; SUVR = standardized uptake value ratio; T = tau.

Abbreviations: A, amyloid; CU, cognitively unimpaired; *GFAP*, glial fibrillary acidic protein; MCI, mild cognitive impairment; MMSE, mini-mental state examination; *n*, number of subjects; SUVR, standardized uptake value ratio; T, tau.

For the subsample of subjects with a follow-up neuropsychological assessment, the average MMSE score was 24 ± 5, with an average rate of change of 1 ± 2 MMSE points per year. A significant difference between MMSE scores was found at baseline and follow-up (*P* < 0.01). When stratifying subjects by AT profile, only the A+T− and A+T+ groups showed significantly different MMSE scores at follow-up when compared with baseline (*P* < 0.01). No significant differences in age or sex were found between the declining group of subjects and the stable individuals.

### Correlation analyses and multilinear regressions at baseline

**Figure 1:**
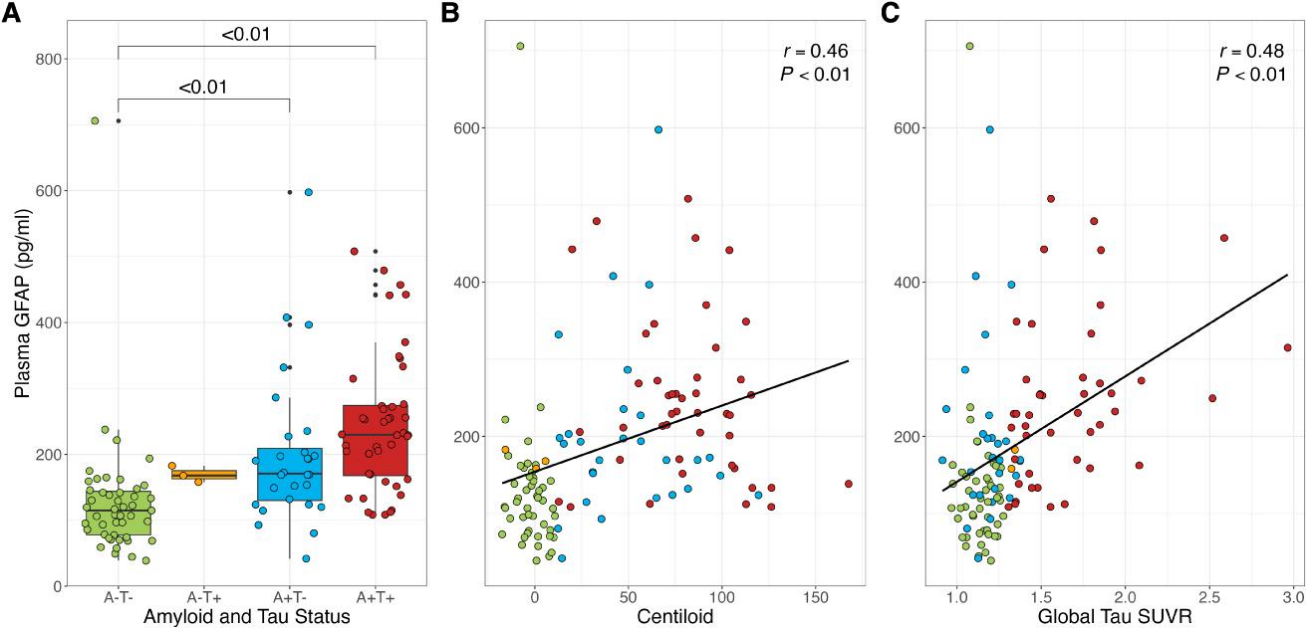
**Distribution of plasma *GFAP* by AT status and its correlation with AT biomarkers**. (**A**) Box plots containing the distribution of plasma glial fibrillary acidic protein (*GFAP*) levels by AT status. Boxes represent the interquartile range of values; the horizontal line indicates the median score per group; whiskers expand up to 1.5 times the interquartile range; remaining dots indicate outliers. Coloured circles represent individual values. Significant differences between groups are marked by a horizonal square bracket with respective *P*-values. (**B**) A scatter plot showing the correlation between Centiloid and plasma *GFAP* values. (**C**) A scatter plot showing the correlation between global tau standardized uptake value ratio (SUVR) and plasma *GFAP* levels. In both scatter plots (**B** and **C**), the solid line represents the linear regression between variables.

Figure 1 shows the difference in *GFAP* values across AT profiles. The correlation between Centiloid and *GFAP* values was significant (Table 2). However, when stratifying subjects by AT profile, the correlation was not significant for any of the profiles (Supplementary Table 1). The correlation between global tau SUVR and *GFAP* levels was also significant (Table 2). When stratifying subjects by AT profile, only the A+T+ subjects showed a significant correlation between variables (*r* = 0.45, *P* < 0.01; Supplementary Table 1). Regional tau SUVR values were also significantly correlated with *GFAP* levels, with the exception of Braak VI (Table 2). When stratified by AT profile, only the A+T+ group showed significant results (Braak III: 0.37, *P* = 0.01; Braak IV: 0.34, *P* = 0.02; Braak V: 0.30, *P* = 0.04), with Braak I/II and VI not showing significant correlations for any of the profiles (Supplementary Table 1). Cortical thickness (*r* = −0.34, *P* < 0.01) and baseline MMSE scores (*r* = −0.34, *P* < 0.01) showed an inverse correlation with *GFAP* levels (Table 2), but when dividing subjects by AT profile, no significant correlations were found (Supplementary Table 1). Multivariate linear regression showed a significant positive association between plasma *GFAP* levels and age (β = 4.1, *P* < 0.01), global tau SUVR (β = 89.4, *P* = 0.01) and cortical thickness (β = −119.9, *P* = 0.04). The remaining variables (Centiloid, sex, education and *APOE* carriership) were not significantly associated with *GFAP*.

**Table 2:** Correlation coefficients of Alzheimer's disease biomarkers or MMSE score with plasma *GFAP* levels

Biomarker	Correlation coefficient	*P*-value
Centiloid	0.46	<0.01
Global VOI tau SUVR	0.48	<0.01
Braak I/II VOI tau SUVR	0.22	0.02
Braak III VOI tau SUVR	0.46	<0.01
Braak IV VOI tau SUVR	0.44	<0.01
Braak V VOI tau SUVR	0.35	<0.01
Braak VI VOI tau SUVR	0.17	0.06
Composite Alzheimer's disease cortical thickness signature	−0.34	<0.01
Baseline MMSE score	−0.34	<0.01

Spearman correlation coefficients of Alzheimer's disease imaging biomarkers or MMSE score with plasma *GFAP* levels at baseline. GFAP = glial fibrillary acidic protein; MCI = mild cognitive impairment; MMSE = Mini-Mental State Examination; SUVR = standardized uptake value ratio; VOI = volume of interest.

Abbreviations: *GFAP*, glial fibrillary acidic protein; MMSE, mini-mental state examination; SUVR, standardized uptake value ratio; VOI, volume of interest.

Significant differences in tau PET SUVR uptake between right and left hemispheres were found for the global and Braak III, IV and VI VOIs, with the left hemisphere showing a bigger uptake. When correlating plasma *GFAP* levels with tau PET SUVR uptake by right and left hemispheres separately, similar results were found to those for the bilateral VOIs. Significant correlations were found for the global (right: *r* = 0.38, *P* < 0.01; left: *r* = 0.40, *P* < 0.01), Braak III (right: *r* = 0.38, *P* < 0.01; left: *r* = 0.38, *P* < 0.01), Braak IV (right: *r* = 0.35, *P* < 0.01; left: *r* = 0.39, *P* < 0.01), Braak V (right: *r* = 0.33, *P* < 0.01; left: *r* = 0.34, *P* < 0.01) and Braak VI left (*r* = 0.21, *P* < 0.01) VOIs. Braak I/II (right: *r* = 0.11, *P* = 0.22; left: *r* = 0.15, *P* = 0.11) and Braak VI right (*r* = 0.17, *P* = 0.06) VOIs were not significantly correlated with plasma *GFAP*.

### Topographical association between tau SUVR and *GFAP*

**Figure 2:**
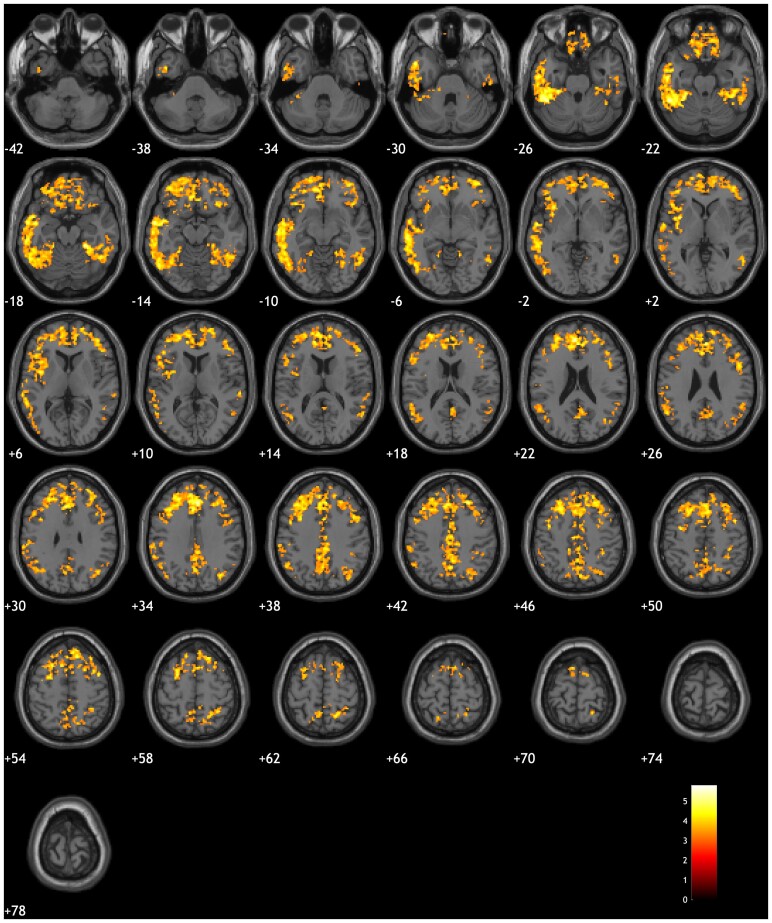
**Voxel-wise association between tau and *GFAP*.** Association between plasma glial fibrillary acidic protein (GFAP) and tau PET standardized uptake value ratio uptake independently of Centiloid. Statistical parametric maps were investigated at *P* < 0.001 with family-wise error-corrected at cluster level. Age, sex, years of education and *APOE* carriership were used as covariates in the model.

The hypothesis that plasma *GFAP* is associated with greater tau PET uptake independently of amyloid burden (measured through Centiloid values) was tested using a voxel-wise multilinear regression model. The results revealed that plasma *GFAP* was associated with increased tau PET SUVR values in the lateral temporal and frontal regions of the brain (false discovery rate corrected at *P* < 0.01; significant clusters: β = 0.001), with the left side of the brain showing higher correlations than the right (Fig. 2). These results were independent of age, sex, education, amyloid burden and *APOE* genotype. The association between tau PET uptake and plasma *GFAP* levels did not change significantly when including baseline MMSE score as a covariate (Supplementary Fig. 1). No clusters were found to be significantly correlated with *GFAP* for any of the amyloid radiotracers.

### Correlation analysis and multilinear regressions at follow-up

At baseline, all imaging biomarkers and plasma *GFAP* levels were significantly correlated with MMSE scores (Supplementary Table 2). MMSE annual rate of change was significantly correlated with Centiloid, global tau SUVR, cortical thickness and plasma *GFAP* levels (Table 3). When dividing tau uptake by Braak regions, only the uptake in Braak VI region was not significantly correlated with the annual rate of MMSE change (Table 3). When separating subjects into AT profiles, the A+T+ group presented significant correlations between annual rate of MMSE change and global tau SUVR (*r* = 0.5, *P* < 0.01), Braak III (*r* = 0.37, *P* = 0.04), Braak IV (*r* = 0.48, *P* < 0.01), cortical thickness (*r* = −0.55, *P* < 0.01) and plasma GFAP (*r* = 0.37, *P* = 0.05), but not with Centiloid, Braak I/II, Braak V and Braak VI. A−T−, A−T+ and A+T− subjects did not present significant correlations for Centiloid, global tau SUVR, Braak regional SUVR, cortical thickness and plasma *GFAP*. Multivariate linear regression showed a significant positive association between MMSE annual rate of change and global tau SUVR (β = 3.24, *P* < 0.01), plasma *GFAP* (β = 0.005, *P* < 0.01) and cortical thickness (β = −2.43, *P* = 0.04). Wilcoxon test showed that plasma *GFAP* levels were significantly higher in individuals who declined cognitively than in the ones who did not in the whole sample (*P* < 0.01; Supplementary Fig. 2A) and only for the A−T− and A+T+ profiles (Supplementary Fig. 2B).

**Table 3:** Correlation coefficients of Alzheimer's disease biomarkers or *GFAP* levels with MMSE annual rate of change

Biomarker	Correlation coefficient	*P*-value
Centiloid	0.41	<0.01
Global VOI tau SUVR	0.43	<0.01
Braak I/II VOI	0.27	<0.01
Braak III VOI	0.47	<0.01
Braak IV VOI	0.44	<0.01
Braak V VOI	0.33	<0.01
Braak VI VOI	0.13	0.20
Composite Alzheimer's disease cortical thickness signature	−0.38	<0.01
Plasma *GFAP*	0.46	<0.01

Spearman correlation coefficients of Alzheimer's disease imaging biomarkers or plasma *GFAP* levels with MMSE annual rate of change. GFAP = glial fibrillary acidic protein; MCI = mild cognitive impairment; MMSE = Mini-Mental State Examination; SUVR = standardized uptake value ratio; VOI = volume of interest.

Abbreviations: *GFAP*, glial fibrillary acidic protein; MMSE, mini-mental state examination; SUVR, standardized uptake value ratio; VOI, volume of interest.

### Mediation analysis

**Figure 3:**
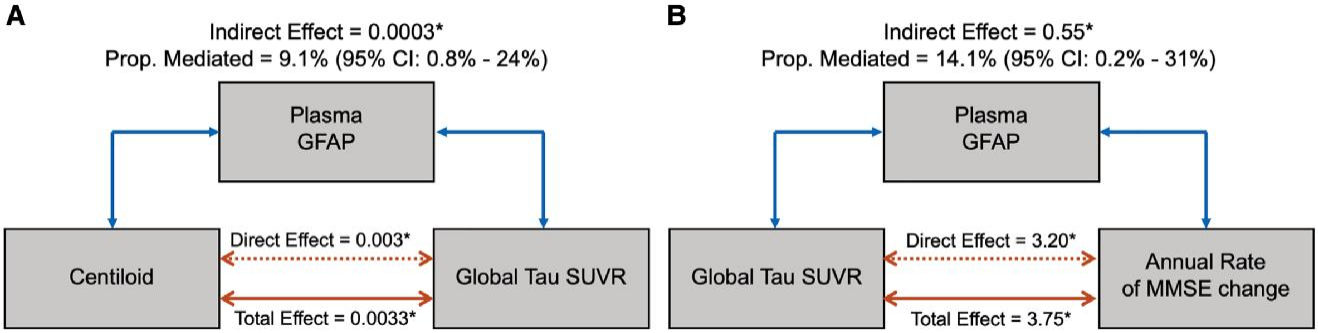
**Mediation analysis results.** Path diagrams indicate whether plasma glial fibrillary acidic protein (*GFAP*) mediated the association between Centiloid and global tau standardized uptake value ratio (SUVR) (**A**) or between global tau SUVR and the annual rate of Mini-Mental State Examination (MMSE) change (**B**), adjusted for age, sex, education, cortical thickness and MMSE scores (**A**) or Centiloid (**B**). The direct effect reflects the extent to which global tau SUVR (**A**) or annual rate of MMSE change (**B**) changes when baseline Centiloid (**A**) or global tau SUVR (**B**) increases by one unit while baseline plasma *GFAP* remains unaltered. The indirect effect reflects the extent to which global tau SUVR (**A**) or annual rate of MMSE change (**B**) changes when baseline Centiloid (**A**) or global tau SUVR (**B**) is held constant and plasma *GFAP* levels change by the amount it would have changed had baseline Centiloid (**A**) or global tau SUVR (**B**) increased by one unit. The total effect is the sum of direct and indirect effects. Asterisks mark statistically significant values.

Figure 3A shows path diagrams assessing plasma *GFAP* as a potential mediator of the associations between Centiloid and global tau SUVR. A statistically significant mediation effect was found [9.1% (95% confidence interval: 0.8%–24%) of the total effect, *P* = 0.02]. Mediation effects of plasma *GFAP* in the association between global tau SUVR and baseline MMSE scores were not significant (*P* = 0.24), whereas the direct effects were (−5.08, *P* < 0.01). Mediation effects of plasma *GFAP* in the association between Centiloid and baseline MMSE were not significant (*P* = 0.08), whereas the direct effects were (−0.02, *P* < 0.01). When assessing the mediation of plasma *GFAP* (Fig. 3B) in the association between global tau SUVR and the annual rate of MMSE change, a statistically significant mediation effect was also found [14.1% (95% confidence interval: 2.2%–31%) of the total effect, *P* = 0.01]. When assessing the mediation of plasma *GFAP* in the association between Centiloid and the MMSE annual rate of change, no significant effects were found.

Mediation analysis by Braak region SUVR instead of global tau PET SUVR showed that plasma *GFAP* mediated the effects of Centiloid in regional tau SUVR in Braak III [12.2% (95% confidence interval: 1.1%–33%) of the total effect, *P* = 0.04], Braak IV [10.0% (95% CI: 1.6%–26%) of the total effect, *P* = 0.02] and Braak V [13.8% (95% confidence interval: 1.9%–36%)] of the total effect, *P* = 0.01), but not in Braak I/II (*P* = 0.94; direct effect = 0.0006, *P* < 0.01) and Braak VI (*P* = 0.12; direct effect = 0.0003, *P* < 0.01).

## Discussion

The main goal of this study was to evaluate the association between Alzheimer's disease pathology measured by PET and plasma *GFAP* concentration as a measure of neuroinflammation in a memory clinic cohort. To this end, an investigation of the association between amyloid and tau PET SUVR and plasma *GFAP* was performed at both regional and voxel levels. In general, plasma *GFAP* was associated with tau deposition mainly in the temporal and inferior frontal lobes, with stronger correlations on the left side of the brain. Furthermore, neuroinflammation measured as GFAP was found to have a partial mediation effect in the studied associations between Centiloid values and tau PET SUVR and with the annual rate of MMSE change globally.

Alzheimer's disease pathology is known to trigger a neuroinflammatory process in the brain that results not only in activated microglia that cannot phagocytose amyloid deposits, leading to plaque accumulation,^49,50^ but also to astrocytic changes in the blood–brain barrier that further impair plaque clearance from the brain.^51^ Therefore, neuroinflammation associated with Alzheimer's disease pathology might be of greater influence than previously considered. The inclusion of plasma *GFAP* as a marker of inflammation in most recent revisions of the amyloid-tau-neurodegeneration (ATN) profile classification is an initial step for further understanding the complex interplay of the response of the brain to pathological deposits.

*GFAP* levels can be measured not only in plasma but also in CSF samples. Previous studies have found that both are markers of neuroinflammation, and measures are correlated, although *GFAP* levels behave differently at each stage of the Alzheimer's disease spectrum when measured using different assays.^12,21^ It has also been suggested that although plasma *GFAP* reflects neuroinflammation caused by reactive astrogliosis attributable to amyloid deposits, CSF *GFAP* is associated with the astrocyte response to neuroinflammatory changes.^21^ Finally, a previous study has found that CSF is an unreliable method to measure *GFAP* in Alzheimer's disease, whereas plasma *GFAP* is a stable matrix.^52^ Therefore, caution must be taken when comparing results of studies with different *GFAP* measuring methods.

When binarizing subjects according to biomarker positivity in AT profiles, it is possible to observe that individuals without the presence of Alzheimer's disease pathology have significantly lower plasma *GFAP* levels than patients with Alzheimer's disease pathology. However, no difference was found between A+T− and A+T+ groups, in line with previous results and suggesting that an increase in *GFAP* represents an early event in Alzheimer's disease pathogenesis.^12^ Although results in the previous section agree with the strong correlation between plasma *GFAP* and amyloid pathology, it was also found that amyloid PET distribution was not significantly correlated with *GFAP* at a voxel level when corrected for other covariates. Furthermore, the correlation between *GFAP* levels and PET biomarkers was significant in general, but it is interesting to notice that when stratifying by AT profile, tau PET uptake remained significantly correlated with *GFAP* levels, suggesting that plasma *GFAP* is not only associated with amyloid deposition, in contrast to what has been suggested by previous studies.^12,53,54^ Finally, in agreement with a previous study, plasma *GFAP* was more strongly correlated with longitudinal cognitive decline than measurements of brain atrophy.^55^ It is important to point out that the association between plasma *GFAP* and tau PET was independent of age, sex, education, MMSE, Centiloid and cortical thickness.

The threshold choice for amyloid deposition in this study was based on previous literature that matches the local sample at the Geneva Memory Clinic. Nonetheless, other thresholds have been suggested in the literature, and these choices are mostly related to the end point of the study being performed. Although lower threshold points, such as the one used in this study, perform well in prevention studies^56^ and seem to be a better fit for *APOE4* carrier patient selection for anti-amyloid studies,^57^ higher thresholds usually have the best agreement with neuropathological and clinicopathological evidence of Alzheimer's disease.^58^ Therefore, the selection of Centiloid threshold for amyloid status binarization should be assessed carefully, taking into consideration the study design and primary end point.

The association between neuroinflammation and global tau PET uptake as a marker has been investigated previously. However, regional SUVR values have shown different association strengths and significance between biomarkers. The correlation between plasma *GFAP* and regional tau PET SUVR being present in only specific regions further supports the use of it as a potential staging biomarker when combined with amyloid and tau. Moreover, plasma *GFAP* was significantly associated with cognitive decline, independently of demographic and pathological characteristics, further promoting its use to assess individual prognosis. However, it is important to mention that elevated levels of *GFAP* have been reported consistently in other neurodegenerative diseases. Indeed, a combination with other biomarkers seems to be an essential condition for the putative use of *GFAP* as a biomarker in Alzheimer's disease.^26^ Although our finding that global tau SUVR is significantly correlated with cognitive decline is in line with previous studies,^59^ this also supports the hypothesis that assessing regional tau uptake instead might offer a better prognostic value of disease progression. However, that remains to be corroborated by future studies.

The topographical association between plasma *GFAP* levels and tau SUVR distribution further highlights the importance of considering regional PET uptake in favour of global values. A lateralized association was found (Fig. 2) between markers, which could be related to the asymmetric and heterogeneous brain distribution of tau aggregates.^60^ Furthermore, a lateralization of tau PET SUVR uptake was also found, with significant differences between the right and left hemispheres in some brain regions. Previous studies have found that brain structure changes throughout the Alzheimer's disease continuum in a lateralized direction, with the left side of the brain being more affected than the right, especially in the temporal lobe.^61-63^ This larger atrophy in the left temporal lobe affects the functional connectivity of this region to the rest of the brain. Given that it has been already shown that the loss of functional connectivity is correlated with a larger tau accumulation,^64^ one might expect a larger correlation between neuroinflammation, as a result of Alzheimer's disease pathology, and tau aggregates in the left hemisphere. A stronger correlation between tau aggregation and neuroinflammation was localized mainly in regions known for typical Alzheimer's disease accumulation. This raises the question of whether different correlation patterns could be found for other tauopathies that can also be studied using tau PET imaging.^65,66^

Previous studies have suggested that plasma *GFAP* could be used as an earlier marker than tau PET in hypothetical models of Alzheimer's disease progression.^12,26^ Results in the previous section concur with these results by showing that *GFAP* mediates the effect of amyloid deposition on tau pathology, in line also with earlier studies concluding that astrocytic activation could facilitate tau pathology spread. Mediation analysis further results in only partial mediation effects, indicating the possible presence of other factors that could mediate the studied effects, such as other markers of neuroinflammation (i.e. microglial activation) and genetic factors (i.e. apolipoprotein *E*4 carriership).

Current disease-modifying clinical trials mostly include the use of anti-amyloid drugs.^67-69^ However, future clinical trials targeting tau aggregates are expected to emerge in the coming years.^70^ It will be important to take into consideration the neuroinflammatory effects of tau pathology in the brain. A possible combination with anti-inflammatory therapies might be of advantage to improve results.

Conclusions from this study are encouraging; however, some limitations need to be pointed out. Firstly, the annual rate of MMSE change was used as a measure for cognitive decline, although MMSE is a global measure characterized by a ceiling effect, being less sensitive in comparison to other neuropsychological tests. Secondly, given that this cohort is a sample from a memory clinic, it is enriched in subjects with higher levels of cognitive decline, which also tend to progress at a faster rate. However, the inclusion of a clinical population in this study can also be considered a strength, because it can more easily translate results into clinical practice. Finally, some subgroups depending on the classification used had a low number of subjects (e.g. subjects visually classified in Braak stage VI or the A−T+ population), which could have prevented significant results in subgroup analysis.

## Conclusion

Elevated plasma *GFAP* levels are associated with increased tau deposition in lateral temporal and frontal regions and with accelerated cognitive decline, independently of tau and amyloid load. *GFAP* also explains, in part, the effect of amyloid pathology on tau accumulation and of tau pathology on subsequent cognitive decline. These results support neuroinflammation and astrogliosis as relevant contributors to Alzheimer's disease pathogenesis, which can be monitored through blood sampling, and suggest neuroinflammation as a potential target for future disease-modifying therapeutic trials targeting tau pathology.

## Supplementary Material

awae211_Supplementary_Data

## Data Availability

The data that support the findings of this study are available from the corresponding author, upon reasonable request.
