# Supplementary material for: Association of glial fibrillary acid protein, Alzheimer's disease pathology and cognitive decline
Source: Brain. 2024 Jun 28;147(12):4094–104. doi: 10.1093/brain/awae211 (PMC11629700; doi:10.1093/brain/awae211)
Supplement: awae211_Supplementary_Data [file awae211_supplementary_data.pdf]

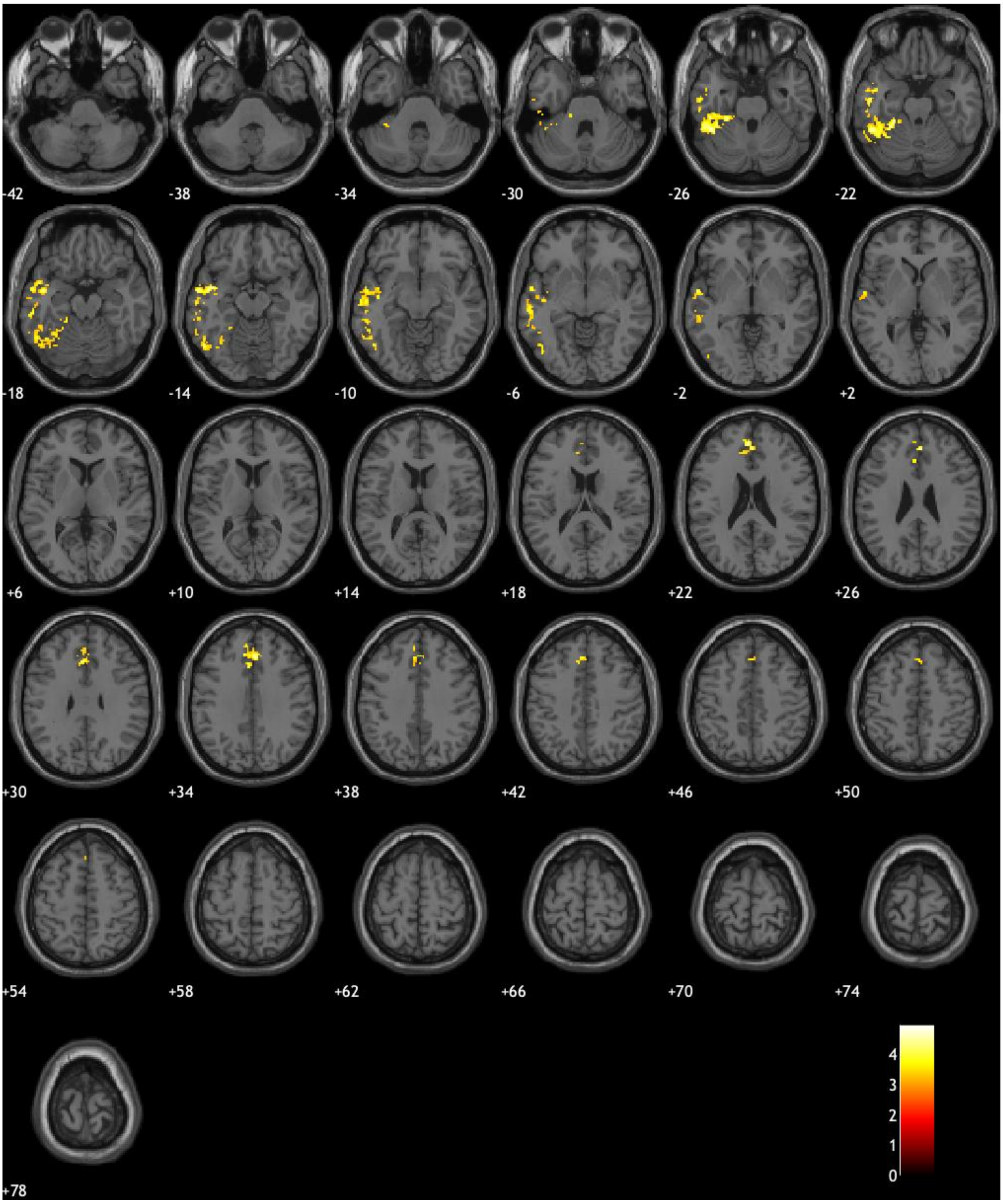

Supplementary Figure 1. Voxel-wise association between tau and GFAP independent of MMSE. Association between plasma GFAP and tau PET SUVR uptake independently of Centiloid. Statistical parametric maps were investigated at $P < 0.001$ with FWE-corrected at cluster level. Age, gender, years of education, APOE carriership, and MMSE score were used as covariates in the model.

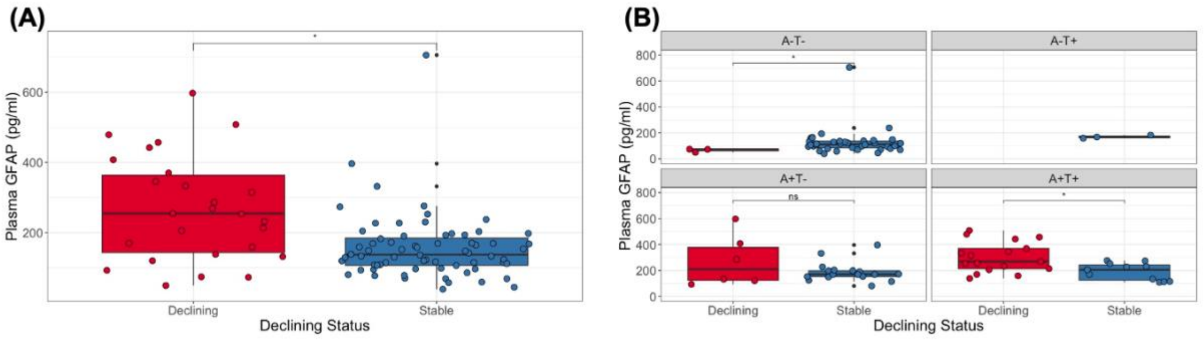

Supplementary Figure 2. GFAP levels by cognitive decline status. Distribution of plasma GFAP levels for subjects that declined (red) or were stable (blue) at follow-up (A) for the complete cohort and (B) by AT status. Declining subjects were defined as subjects with an annual MMSE rate of change larger than 1, while stable individuals presented a rate smaller than 1. Boxes represent the interquartile range of values; the horizonal line, the median score per group; the whiskers expand up to 1.5 times the interquartile range; and the remaining black dots correspond to outliers. Coloured circles represent individual values.

* $P < 0.05$

GFAP = glial fibrillary acid protein, pg = picogram, ml = millilitre, A = amyloid, T = tau

Supplementary Table 1. Correlation of Alzheimer's disease biomarkers or MMSE with plasma GFAP by AT profile.

Biomarker	A-T-	A-T+	A+T-	A+T+
Centiloid	-0.27	-0.50	-0.02	-0.15
Global VOI Tau SUVR	-0.04	0.50	-0.07	0.45*
Braak I/II VOI Tau SUVR	-0.10	0.50	-0.10	-0.01
Braak III VOI Tau SUVR	-0.08	0.50	-0.06	0.37*
Braak IV VOI Tau SUVR	-0.13	-0.50	-0.12	0.34*
Braak V VOI Tau SUVR	-0.10	1	-0.19	0.30*
Braak VI VOI Tau SUVR	-0.13	1	-0.28	0.23
Composite AD Cortical Thickness Signature	-0.18	0.50	-0.10	-0.26
Baseline MMSE score	0.10	-0.50	-0.30	-0.28

Spearman correlation coefficients of Alzheimer's disease imaging biomarkers or MMSE score with plasma GFAP levels at baseline by AT profile.

* *P*-value > 0.05

VOI = Volume of Interest, SUVR = Standardised Uptake Value Ratio, AD = Alzheimer's Disease, MMSE = Mini-Mental State Examination

Supplementary Table 2. Correlation of AD biomarkers or GFAP with MMSE at baseline.

Biomarker	Correlation Coefficient	*P*-value
Centiloid	-0.45	< 0.01
Global VOI Tau SUVR	-0.48	< 0.01
Braak I/II VOI Tau SUVR	-0.31	< 0.01
Braak III VOI Tau SUVR	-0.48	< 0.01
Braak IV VOI Tau SUVR	-0.44	< 0.01
Braak V VOI Tau SUVR	-0.41	< 0.01
Braak VI VOI Tau SUVR	-0.29	< 0.01
Composite AD Cortical Thickness Signature	0.32	< 0.01
Plasma GFAP	-0.34	< 0.01

Spearman correlation coefficients of Alzheimer's disease imaging biomarkers or plasma GFAP levels with MMSE scores at baseline.

VOI = Volume of Interest, SUVR = Standardised Uptake Value Ratio, AD = Alzheimer's Disease, MMSE = Mini-Mental State Examination
